# Susceptibility to Infection and Impact of COVID-19 Vaccines on Symptoms of Women with Endometriosis: A Systematic Review and Meta-Analysis of Available Evidence

**DOI:** 10.1007/s43032-024-01707-4

**Published:** 2024-09-27

**Authors:** Gaetano Riemma, Andrea Etrusco, Antonio Simone Laganà, Marco Torella, Maria Giovanna Vastarella, Luigi Della Corte, Antonio D’Amato, Marco La Verde, Pasquale De Franciscis, Luigi Cobellis

**Affiliations:** 1 Department of Woman, Child and General and Specialized Surgery, University of Campania “Luigi Vanvitelli”, Largo Madonna Delle Grazie, 80128 Naples, Italy; 2 Department of Health Promotion, Mother and Child Care, Internal Medicine and Medical Specialties (PROMISE), University of Palermo, 90127 Palermo, Italy; 3 Unit of Obstetrics and Gynecology, “Paolo Giaccone” Hospital, 90127 Palermo, Italy; 4 Department of Neuroscience, Reproductive Sciences and Dentistry, School of Medicine, University of Naples Federico II, 80131 Naples, Italy; 5 Department of Interdisciplinary Medicine (DIM), Unit of Obstetrics and Gynecology, University of Bari “Aldo Moro”, Policlinico of Bari, Bari, Italy

**Keywords:** Endometriosis, COVID-19, SARS-CoV-2, Vaccination, Symptoms, Susceptibility, Dysmenorrhea, Pelvic pain

## Abstract

Women with endometriosis were deemed more prone to COVID-19 infection in some reports. Considering that endometriosis-related aberrant immune response, understanding how COVID-19 vaccination influences its clinical status is crucial. The aim of this meta-analysis was the evaluate the susceptibility to COVID-19 infection and modifications of symptoms following COVID-19 vaccination in women with endometriosis. Electronic searches on EMBASE, MEDLINE, Scopus, Cochrane at CENTRAL, Scielo.br, LILACS and other databases were searched from inception to March 2024. Studies were eligible if they analyzed the incidence of infection in endometriosis women or the changes in symptoms after two doses of COVID-19 vaccine and had a control group. Four studies (2249 women) were included. No increased susceptibility to COVID-19 infection due to presence or absence of endometriosis was retrievable (risk ratio (RR) 1.42 [95% CI 0.88 to 2.27]; I^2^ = 33%). Patients with endometriosis did not experience an overall worsening of symptomatology relative to controls (RR 1.58 [95% CI 0.67 to 3.75]; I^2^ = 94%). An increase in the risk of dysmenorrhea worsening was noted (RR 1.88 [95% CI 1.11 to 3.17]; I^2^ = 63%). No other differences regarding menstrual flow (RR 1.25 [95% CI 0.70 to 2.23]; I^2^ = 78%), intermenstrual bleeding (RR 1.14 [95% CI 0.83 to 1.56]; I^2^ = 39%) and pelvic pain (RR 2.55 [95% CI 0.65 to 10.05]; I^2^ = 80%) compared to controls was retrievable. Therefore, mRNA vaccines do not seem to lead to worsening of symptomatology in endometriotic women. However, a slight temporary increase in dysmenorrhea may be present. Moreover, endometriosis does not seem to increase the risk of contracting COVID-19.

**Supplementary Information:**

The online version contains supplementary material available at 10.1007/s43032-024-01707-4.

## Introduction

Ten to fifteen percent of women who are of reproductive age suffers from endometriosis [1], a common disorder characterized by the presence of endometrial-like glands and stroma out of the uterus that typically manifests as perimenstrual or cyclical symptoms including dysmenorrhea, ovulation discomfort, and irregular bleeding [2].

Recently, it has been proposed that endometriosis may influence a person's vulnerability to COVID-19 [3]. The rationale is related to the peritoneal fluid of endometriosis-afflicted patients has been discovered to include high concentrations of angiogenic factors and inflammatory cytokines, such as IL-1β, IL-6, and IL-10, inducing the synthesis of VEGF (vascular endothelial growth factor) and MCP-1 (monocyte chemotactic protein) by macrophages [4–6]. This results in a pro-inflammatory state that is marked by neutrophil and macrophage activation as well as a decrease in natural killer cell activity [7].

Similarly, infection with SARS-CoV-2 is linked to an uncontrolled immune response known as a "cytokine storm," which is defined by a rise in the levels of pro-inflammatory cytokines including IL-1β and IL-6 as well as chemokines like CXCL10 and CCL2.

Given the immunological similarities between COVID-19 and hyperinflammatory disorders, it is plausible that endometriosis and SARS-COV-2 infection could be positively correlated [8–10].

On the other hand, given that women with endometriosis might have already been significantly impacted by the COVID-19 pandemic, some studies investigated about the possible effects of the SARS-CoV-2 immunization to plan and recommend specific care for these patients [11]. Similarly, there have been multiple questions in recent years regarding the use of hormone medications in conjunction with the COVID-19 immunization.

Several recent studies have assessed menstruation-related problems linked to the SARS-CoV-2 immunization [12]; these studies have reported irregularities in monthly patterns, including dysmenorrhea, heavy menstrual bleeding, or intermenstrual spotting [5, 13]. On the contrary, other studies did not report a plausible impact related to the vaccination. Moreover, these menstrual changes were mostly transient and were not characterized as chronic or harmful [14]. In addition, earlier research on hormone dysfunction on menstruation-related issues after immune stimulation, like the human papillomavirus vaccination, revealed no correlation between the vaccine and symptoms or the ovarian failure reported afterward [15].

However, the interplay between vaccination-induced immune responses and autoimmune conditions, such as endometriosis, is still unknown. Given that endometriosis involves an aberrant immune response, understanding how COVID-19 vaccination influences this dynamic is crucial.

Therefore, the aim of this systematic review and meta-analysis was the analyze the susceptibility to COVID-19 infection of women with endometriosis and to evaluate changes in the severity and nature of endometriosis symptoms following COVID-19 vaccination.

## Methods

This meta-analysis followed the guidelines of the Preferred Reporting Items for Systematic Reviews and Meta-Analyses (PRISMA) [16]. The study protocol was defined a-prior and outlined the search and selection of literature, criteria for article inclusion and exclusion, methodologies for data analysis, and statistical techniques. It was registered in the International Prospective Register of Systematic Reviews (PROSPERO) (CRD42024531947).

### Data Collection and Search Methodology

The research utilized multiple electronic databases such as EMBASE, MEDLINE (accessed via PubMed), Scopus, Scielo.br, and LILACS, employing the following keywords and MeSH terms: “covid-19” and “(vaccine or vaccination)” and “endometriosis”, up until March 2024 without date limitations. Additional searches were done in CINAHL, PsycINFO, and AMED to uncover further pertinent studies and mitigate publication bias. Searches were extended to Clinicaltrials.gov, the Cochrane Central Register of Controlled Trials, and the WHO International Clinical Trials Registry Platform to locate more trials. Gray literature sources and conference abstracts were also reviewed, and references of selected studies were examined to identify additional relevant research. The search was unrestricted by language or geography, but commentaries, letters, editorials, and review articles were excluded.

### Study Selection Criteria and Data Extraction

The inclusion criteria were any randomized, prospective, or retrospective studies that included women (aged over 18 years old) diagnosed with a clinical, instrumental or surgical diagnosis of endometriosis vaccinated for COVID-19 with at least two doses of mRNA-based COVID-19 vaccines approved by the European Medicines Agency (EMA) (Spikevax [Moderna] or Comirnaty [Pfizer-BioNTech]). and had a control group consisting of women vaccinated for COVID-19 but with no clinical, instrumental, or surgical diagnosis of endometriosis. Both women with endometriosis and controls were assessed for any worsening of endometriosis-related symptoms.

According to available studies, to avoid any specific comorbidity that could positively or negatively interfere with susceptibility to COVID-19 infection, patients under the age of 18 and over 50, as well pregnant women were excluded. Moreover, women diagnosed with moderate/severe allergic asthma, chronic lung diseases (pulmonary fibrosis, sarcoidosis, tuberculosis, chronic obstructive pulmonary disease, emphysema), diabetes mellitus, hypertension, ischemic heart disease, chronic renal failure, autoimmune disease, cancer or using chronic medical therapies that could compromise the functioning of the immune system, were excluded.

The abstraction forms were created specifically for this meta-analysis. Patient characteristics, study duration, setting, COVID-19 vaccine information, control group features, outcomes investigated, mean follow-up length, results, and quality of evidence analysis were among the important features that were noted.

Independently, two authors (G.R., A.E) screened and categorized each abstract. Two authors individually retrieved relevant data on the research characteristics and the results of interest, and then came to a consensus on plausible relevance. They also independently assessed the full text of the selected papers. The review panel deliberated about each inconsistency and, after discussion with a third author (A.S.L.), an agreement was established. When the research methods stated that additional outcome data were recorded, unpublished data were, if needed, obtained by direct communication with the original study authors.

### Main Outcome Measures

The co-primary outcomes of this meta-analysis were susceptibility to COVID-19 infection (evaluated as incidence of confirmed infections using a positive antibody or real-time polymerase chain reaction swab between population and controls) changes in bleeding pattern during menstruation (worsening in amount or flow of bleeding), intermenstrual bleeding, changes or worsening in dysmenorrhea and chronic pelvic pain.

### Assessment of Risk of Bias

Every included study had its risk of bias assessed using the Newcastle–Ottawa Scale criteria [17]. According to these criteria, the evaluation of the study is predicated on the choice and comparability of the study groups as well as the identification of the desired outcome. The criteria used to choose a study include assessing the exposed cohort's representativeness, selecting the non-exposed cohort, determining exposure, and providing evidence that the desired outcome was unlikely to occur spontaneously at the outset of the study. The comparability of research is assessed by looking at the equivalence of cohorts depending on the design or analysis.

Furthermore, the methods employed to evaluate the follow-up duration, quality, and outcome of interest are utilized to evaluate the effectiveness of the exposure. For every numbered item in the Selection and Outcome categories, a study can receive up to one star on the Newcastle–Ottawa Scale. Two stars is the maximum that may be given for comparability. A maximum score of nine could be given in accordance with the Newcastle–Ottawa Scale standards [17].

### Statistical Analysis

To analyze the data, Review Manager 5.3 (The Nordic Cochrane Centre 2014) was used. After using Der Simonian and Laird’s random-effects model, the summary measures were presented as a risk ratio (RR) or mean difference with a 95% confidence interval (CI). To overcome potential heterogeneity, a Higgins I^2^ index greater than 0% was considered, whereas 25%, 50% and 75% were considered cut-offs for low, intermediate, and high heterogeneity. The potential publication bias was investigated using the visual evaluation of the funnel plot and the Egger test. A *p* value less than 0.05 was considered statistically significant.

## Results

**Fig. 1:**
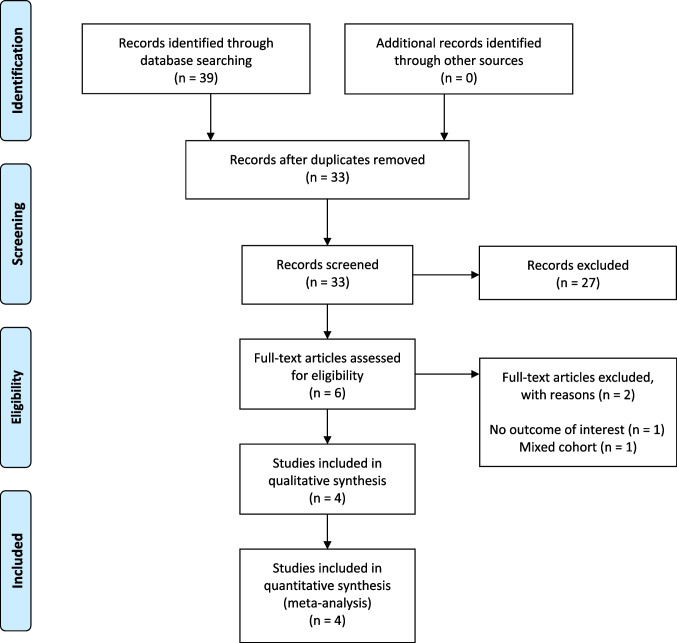
PRISMA Flow-diagram of studies included in systematic review and meta-analysis

The database search yielded 39 studies. After duplicate removal, 33 studies were examined. Of those, after the application of selection criteria, six studies were selected. Among these, one study was removed due to a mixed cohort of women with and without endometriosis, while one was removed for not reporting outcomes of interest. Finally, four studies (summarizing data for 2249 women) were selected for systematic review and meta-analysis [18–21] (Fig. 1).

### Study Characteristics

Studies were all carried out between 2020 and 2022 during the COVID-19 pandemic. All the research, except for one [21], were conducted in high-income countries.

One study was a prospective cohort analysis of women with endometriosis and two doses of mRNA vaccine vs. vaccinated non-endometriotic women [18]. Another study analyzed adverse changes in endometriosis-related symptoms of vaccinated women from the Endometriosis UK mailing list using a questionnaire and subsequently compared to women from the hospital staff without the disease [19]. Two papers focused on analyzing the susceptibility to Covid-19 infections according to presence or absence of a confirmed endometriosis diagnosis [20, 21] (Table 1). All the studies received the approval from their local ethical committees.

**Table 1:** Main characteristics of included studies

Study, Year	Design	Location	Duration	Population	Vaccine	Control	Outcomes	Sample Size
Martinez-Zamora, 2023 [18]	Prospective cohort study	Spain	2021–2022	Endometriosis women > 18 years old who had received at least two doses of vaccine	mRNA-based vaccines	Healthy women with same characteristics and no endometriosis	Changes in a) dysmenorrhea; b) menstrual flow; c) intermenstrual bleeding; pelvic pain; d) overall changes of endometriosis symptoms	848
Gilan, 2023 [19]	Questionnaire-based cross-sectional cohort study	Israel	2021	women with histological diagnosis of endometriosis	mRNA BNT162b2 vaccine	Female medical staff with no endometriosis	Changes in a) dysmenorrhea; b) menstrual flow; c) intermenstrual bleeding; pelvic pain; d) overall changes of endometriosis symptoms	174
Barretta, 2022 [20]	Retrospective case–control study	Italy	2020–2021	Women with histological diagnosis of endometriosis	/	Healthy women without endometriosis complaints	Susceptibility to infection	200
Moazzami, 2021 [21]	Retrospective case–control study	Iran	2020–2021	Women with histological diagnosis of endometriosis	/	Healthy women without endometriosis	Susceptibility to infection	1027

Table 2 reports the inclusion and exclusion criteria for the papers included in the meta-analysis.

**Table 2:** Inclusion and exclusion criteria for included studies

Study, year	Inclusion Criteria	Exclusion Criteria
Martinez-Zamora, 2023 [18]	Women over 18 years old who had received at least two doses of mRNA-based COVID-19 vaccines	Women < 18 years old; menopausal status or receiving GnRH treatment; malignancy; gynecological diseases other than endometriosis; women who did not receive at least two doses of the mRNA COVID-19 vaccines and inability to complete the questionnaire or did not provide informed consent
Gilan, 2023 [19]	Women with a definitive diagnosis of endometriosis who were recruited through hospital records of Endometriosis center	Women who had been previously diagnosed with a COVID-19 infection; women with other gynecological or immunological disorders; pregnant or lactating women
Barretta, 2022 [20]	Women with histologically confirmed endometriosis referred to pelvic pain clinic	NA
Moazzami, 2021 [21]	Women with histopathological diagnosis of endometriosis diagnosed between 1 and 10 years before the study	Patients younger than 18 or older than 45

*NA* not available

### Risk of Bias

Using the Newcastle–Ottawa Scale criteria, all reviewed studies indicated high scores, with values ranging from a minimum of 7 to a maximum of 8. The cohort comparability reached its peak based on controls for age as the main factor and parity as additional factor. Table S1 in the Supplementary Materials provides an extensive point-by-point description of the assessment. Publication bias, evaluated using funnel plot analysis (Figure S1) and Egger’s test (p = 0.723) was not apparent.

### Synthesis of Results

#### Susceptibility to COVID-19 Infection

**Fig. 2:**
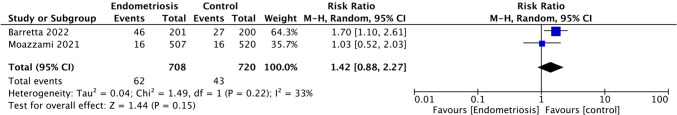
Forest plot for susceptibility to COVID-19 infection in endometriosis women vs. controls

Two studies, providing data for 1428 women, reported the incidence of COVID-19 infections. There was no increased susceptibility to the infection according to the presence or absence of an endometriosis diagnosis (RR 1.42 [95% CI 0.88 to 2.27]; I^2^ = 33%) (Fig. 2).

### Changes in Endometriosis-Related Symptoms

**Fig. 3:**
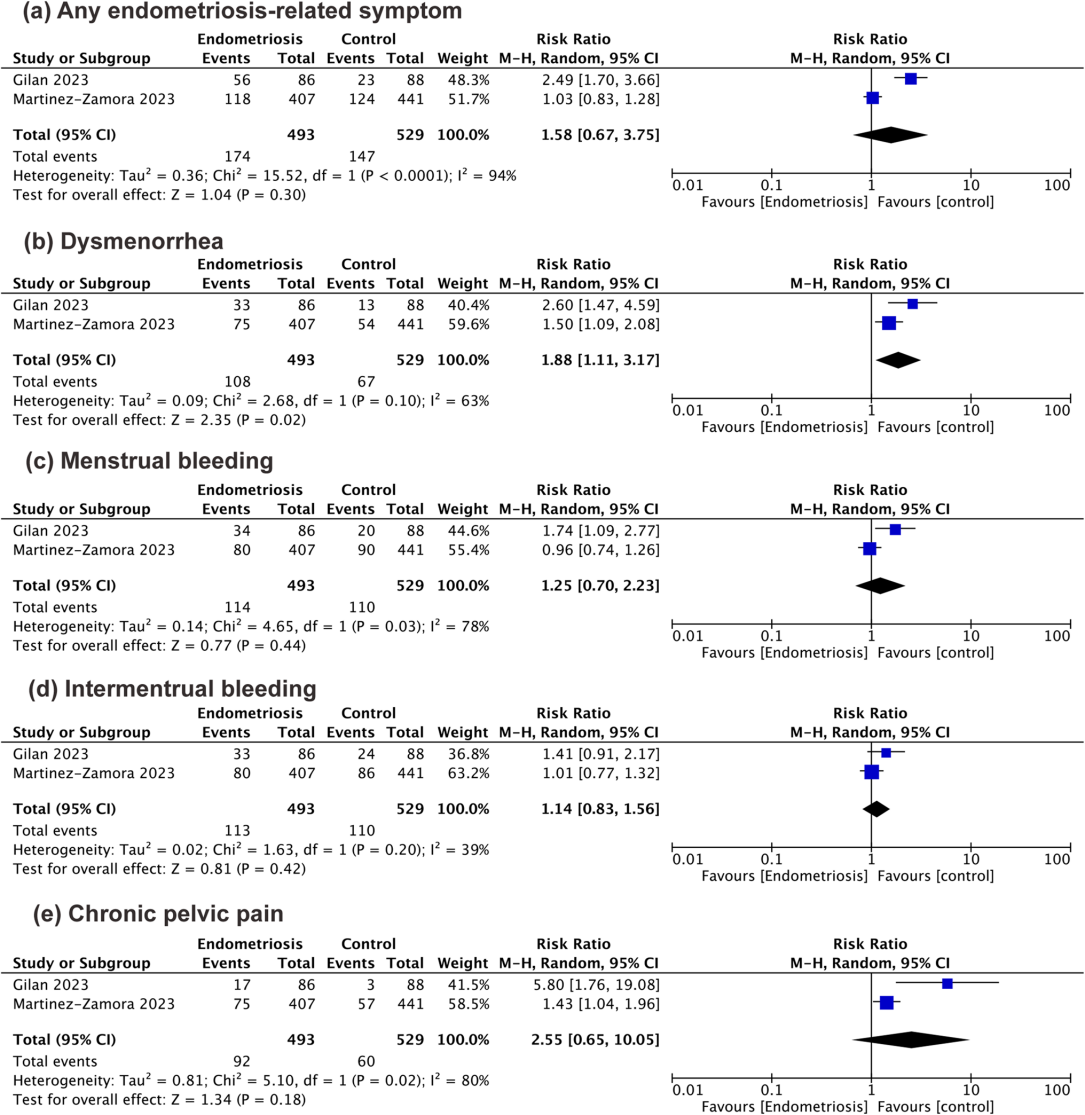
Forest plot for (**a**) any worsening of endometriosis-related symptoms; (**b**) worsening of dysmenorrhea in endometriosis women vs. controls; (**c**) worsening of menstrual bleeding; (**d**) for increased intermenstrual bleeding; (**e**) increased chronic pelvic pain in endometriosis women vs. controls

Overall, two studies reported changes in any of endometriosis-related symptoms in 493 endometriotic women and 529 healthy controls. There was no increased risk for the overall worsening of symptoms (RR 1.58 [95% CI 0.67 to 3.75]; I^2^ = 94%) (Fig. 3a).

Conversely, there was a 1.88-folded increased risk of worsening in patients complaining dysmenorrhea when endometriosis was present (RR 1.88 [95% CI 1.11 to 3.17]; I^2^ = 63%) (Fig. 3b).

The amount of menstrual bleeding was not negatively affected by the mRNA vaccine in both endometriosis women and controls (RR 1.25 [95% CI 0.70 to 2.23]; I^2^ = 78%) (Fig. 3c).

Similarly, patients with endometriosis did not experience increased intermenstrual bleeding compared to healthy controls (RR 1.14 [95% CI 0.83 to 1.56]; I^2^ = 39%) (Fig. 3d).

Moreover, chronic pelvic pain seemed not to be affected by the double-dose vaccination when compared between endometriosis patients and healthy females (RR 2.55 [95% CI 0.65 to 10.05]; I^2^ = 80%) (Fig. 3e).

## Discussion

This systematic review and meta-analysis showed that, overall, the whole symptomatology of women with endometriosis seems not be worsened after two doses of COVID-19 vaccines based on mRNA technology. However, a worsening of dysmenorrhea could happen in women with endometriosis. Nonetheless, women with endometriosis do not seem to be more prone to develop COVID-19 infections compared to healthy controls.

The COVID-19 crisis emerged as a significant global disaster in recent years, transforming the daily routines of people and pushing healthcare infrastructures to adapt swiftly [22]. Within this context, women with non-life-threatening chronic illnesses, such as endometriosis, had to postpone their routine check-ups amid the strictest periods of lockdown, leaving them at risk of feeling overlooked and disconnected [23]. The commercialization of the mRNA COVID-19 vaccine has deeply improved the quality of treatment for these patients [24].

Evidence has shown that changes in menstrual cycles linked to COVID-19 vaccinations are transient, but understanding the effects of vaccinations on menstrual irregularities is crucial [25]. Access to accurate and comprehensive information about these irregularities is essential for managing potential complications, discomfort, and particularly for women who are eager for pregnancy. This is even more critical for women with pre-existing diseases like endometriosis, where sufferers often experience heightened stress, physical symptoms, and are more prone to depression and anxiety. The pandemic has significantly affected the psychological well-being of women with endometriosis, increasing their risk of experiencing post-traumatic stress disorder (PTSD), especially among those already dealing with anxiety, older women, and those who are unemployed. Given the ongoing nature of the pandemic, these insights should encourage healthcare providers to devise effective support strategies for these challenging times, especially for those with chronic conditions. Telehealth services, such as video calls or phone consultations, could offer a viable solution to alleviate feelings of isolation among these women [25].

A plausible etiopathogenetic link for the slight worsening of dysmenorrhea reported in our analysis and in previous studies is yet to be established. Proposed explanations for the observed correlation between the mRNA vaccines and the exacerbation could be linked to the vaccination's effects on the immune and inflammatory systems. According to recent research, several immunologic variables cross-talks triggered by the vaccine may alter myometrial contractility and cervical mucus density [12]. On the contrary, some authors highlighted the psychological and psychosomatic role of vaccine skepticisms in creating false flags in patients’ reported symptoms, including dysmenorrhea, showing that women with more skepticism on the topic had increased chances of reporting worsened menstrual symptoms [26, 27].

Moreover, given the intricate nature of endometriosis and its related symptoms, it should be also noted that several factors might have impacted on the overall findings, especially on dysmenorrhea. Firstly, the role hormone treatment should be taken into consideration. Martinez-Zamora et al. [18] reported that, in women with endometriosis, hormone therapy specifically demonstrated a protective impact on symptoms. During the first and second cycles following immunization, a notably greater proportion of endometriosis patients who were not receiving hormone therapy reported experiencing new or worsened menstrual-related symptoms. On the contrary, in the non-endometriosis group, there were no statistically significant differences between patients receiving hormonal treatment and those who did not [18]. Other factors, including lifestyle habits, dietary pattern, non-hormonal treatments, reduction of physical exercise, were not analyzed in the original studies and deserve further evaluation [22, 28].

A study by Li et al. [29] revealed that menstrual irregularities following COVID-19 infection were short-lived, with most women's cycles normalizing within a few months. This suggests that menstrual changes observed post-vaccination might also be temporary, warranting further investigation in long-term studies. Nonetheless, there's still a debate in scientific literature regarding the impact of COVID-19, associated restrictions, and vaccines on women's health, with menstrual changes also noted in those without endometriosis. Meanwhile, Polese et al. [30] have linked the pandemic-induced psychological stress to menstrual disruptions, particularly highlighting the presence of menstrual irregularities, dysmenorrhea, and premenstrual syndrome among young female medical trainees. It was also found that stress and sleep disturbances during the pandemic significantly affected university students. [30].

On the other hand, Aftab et al.'s findings contrasted, showing no significant change in menstrual cycles among the majority of their study participants post-COVID-19 infection [31].

A systematic analysis of 17799 patients highlighted a 7.5% COVID-19 infection rate among those with endometriosis, with notable health impacts including reduced access to medical care and an increase in symptoms such as dysmenorrhea, anxiety, depression, and fatigue. The pandemic undeniably exacerbated the challenges faced by endometriosis patients, worsening their symptoms and overall well-being [32].

Women previously diagnosed with endometriosis through laparoscopy were found to have a 22% higher chance of experiencing long COVID-19 symptoms, with an even stronger correlation observed for symptoms lasting over eight weeks [33].

However, this meta-analysis and its related studies showed no increased susceptibility to COVID-19 infections when endometriosis is diagnosed. Several authors tried to speculate about the plausible mechanism leading to such issue. A lower risk of endometrial-like tissue, as for endometriotic implants, susceptibility to the viral infection has been linked to the lower expression of host proteins related to SARS-CoV-2, such as Transmembrane protease 2 serine protease-2 (TMPRSS2) and especially angiotensin-converting enzyme 2 (ACE2), which is the entry point on the cell [34]. Moreover, through the synthesis of sex hormones, ACE2 protein has a beneficial role in the physiology, pathophysiology, and fertility processes of the female reproductive system. In women with endometriosis, ACE2 and TMPRSS2 expression is significantly downregulated compared to healthy controls. Therefore, endometrial-like cells could be less prone to be invaded by the virus and such mechanism might have a protective effect against the COVID-19 infection [34].

The COVID-19 pandemic has led to decreased hospital visits, intensified symptoms, longer hospital stays, and diminished life quality for endometriosis patients, likely due to hormonal imbalances triggered by increased psychological stress. These findings underscore the importance of optimizing care for endometriosis patients and prioritizing early psychological interventions during such crises. Therefore, ascertaining that vaccination with mRNA should be considered feasible and safe in most women with endometriosis is important to avoid the consequences of long COVID-19 and reduced quality of life.

Nonetheless, it is important to acknowledge certain limitations associated with this quantitative analysis. Primarily, the findings rely on subjective symptoms and personal experiences of patients, which could be prone to bias. The clinical impressions shared by the women were not confirmed by medical professionals or gynecologists through objective measures. Unfortunately, there is a lack of data on objective metrics in the existing literature, and the subjects assessed the results based on their own perceptions of normalcy from their everyday life experiences before the enrollment in the original studies. Additionally, the limited number of studies included, which significantly increased the heterogeneity of the results for certain outcomes, poses a marked challenge in stating comprehensive conclusions. Moreover, the results may be affected by population bias due to the retrospective design of some of the included papers. Similarly, variations in other traits among the groups might impact the outcomes. Furthermore, participants were recruited using a prospective or retrospective method, and the non-random selection of patients heightens the likelihood of confounding factors affecting the results. Lastly, an additional limitation should be related to the absence of long-term follow-up in all the cohorts analyzed in the quantitative synthesis; therefore, we were not allowed to draw additional considerations on the length and potential duration of worsened dysmenorrhea and the other menstrual symptoms.

This systematic review highlights numerous strengths. Initially, it stands out as the first research effort to quantify data from different endometriosis patients who have received the COVID-19 mRNA vaccine into a meta-analysis. Furthermore, while the study number is small, the cumulative participant total is deemed sufficient to affirm the initial reliability of the findings. Lastly, the analysis benefits from the exclusivity of data derived from individual cohorts, ensuring there is no duplication of data across studies that assessed the same groups of women in national registries (e.g. Endometriosis UK).

## Conclusions

The use of mRNA vaccines for avoiding COVID-19 infection seems not to increase the worsening of overall symptomatology in women with a diagnosis of endometriosis compared to healthy patients. However, a slight increase in dysmenorrhea may be present, even if such symptom should be temporary. Moreover, the presence of endometriosis does not seem to increase the risk of susceptibility to COVID-19 infections. However, the limited number of included studies and its related limitation reduces the overall generalizability of the findings, requiring further clarifications in upcoming research.

## Supplementary Information

Below is the link to the electronic supplementary material.

Supplementary file1 Table S1 Quality scores of the studies included in the meta-analysis, assessed by the Newcastle-Ottawa scale (DOCX 70 KB)

Supplementary file2 Figure S1 Funnel plot for the co-primary outcome (susceptibility to COVID-19 infection) (PDF 19 KB)

## Data Availability

All data generated or analyzed during the present study are included in the published article and its supplementary material file.

## Code Availability

Not applicable for systematic reviews.
