# Supplementary material for: Susceptibility to Infection and Impact of COVID-19 Vaccines on Symptoms of Women with Endometriosis: A Systematic Review and Meta-Analysis of Available Evidence
Source: Reprod Sci. 2024 Sep 27;31(11):3247–56. doi: 10.1007/s43032-024-01707-4 (PMC11527924; doi:10.1007/s43032-024-01707-4)
Supplement: Supplementary file 1 — Supplementary file1 Table S1 Quality scores of the studies included in the meta-analysis, assessed by the Newcastle-Ottawa scale (DOCX 70 KB) [file 43032_2024_1707_MOESM1_ESM.docx]

**Table S1.** Quality scores of the studies included in the meta-analysis, assessed by the Newcastle-Ottawa scale.

|  |  | **Selection** | | | | **Comparability ^a^** | **Outcome** | | | **Overall quality** |
| --- | --- | --- | --- | --- | --- | --- | --- | --- | --- | --- |
| **Author (et al); reference** | **Year of publication** | **Representativeness of the exposed cohort** | **Selection of the non-exposed cohort** | **Ascertainment of exposure** | **Demonstration that the outcome of interest was not present at the start of the study** | **Comparability of cohorts on the basis of the design or analysis** | **Assessment of the outcome** | **Was follow-up enough for outcomes to occur** | **Adequacy of follow-up of cohorts** |  |
| Martinez-Zamora [18] | 2023 | * | * | * | * | * | * | * | * | 8 |
| Gilan, [19] | 2023 | * | * | * | * | * | * | * | * | 8 |
| Barretta [20] | 2022 | 0 | * | * | * | * | * | * | * | 7 |
| Moazzami [21] | 2021 | * | * | * | * | * | * | * | * | 8 |

^a^ Comparability of cohorts: for the most important factor: study controls adjusted for age; additional factor: study controls for parity
