# Supplementary figures and images for: Susceptibility to Infection and Impact of COVID-19 Vaccines on Symptoms of Women with Endometriosis: A Systematic Review and Meta-Analysis of Available Evidence
Source: Reprod Sci. 2024 Sep 27;31(11):3247–56. doi: 10.1007/s43032-024-01707-4 (PMC11527924; doi:10.1007/s43032-024-01707-4)

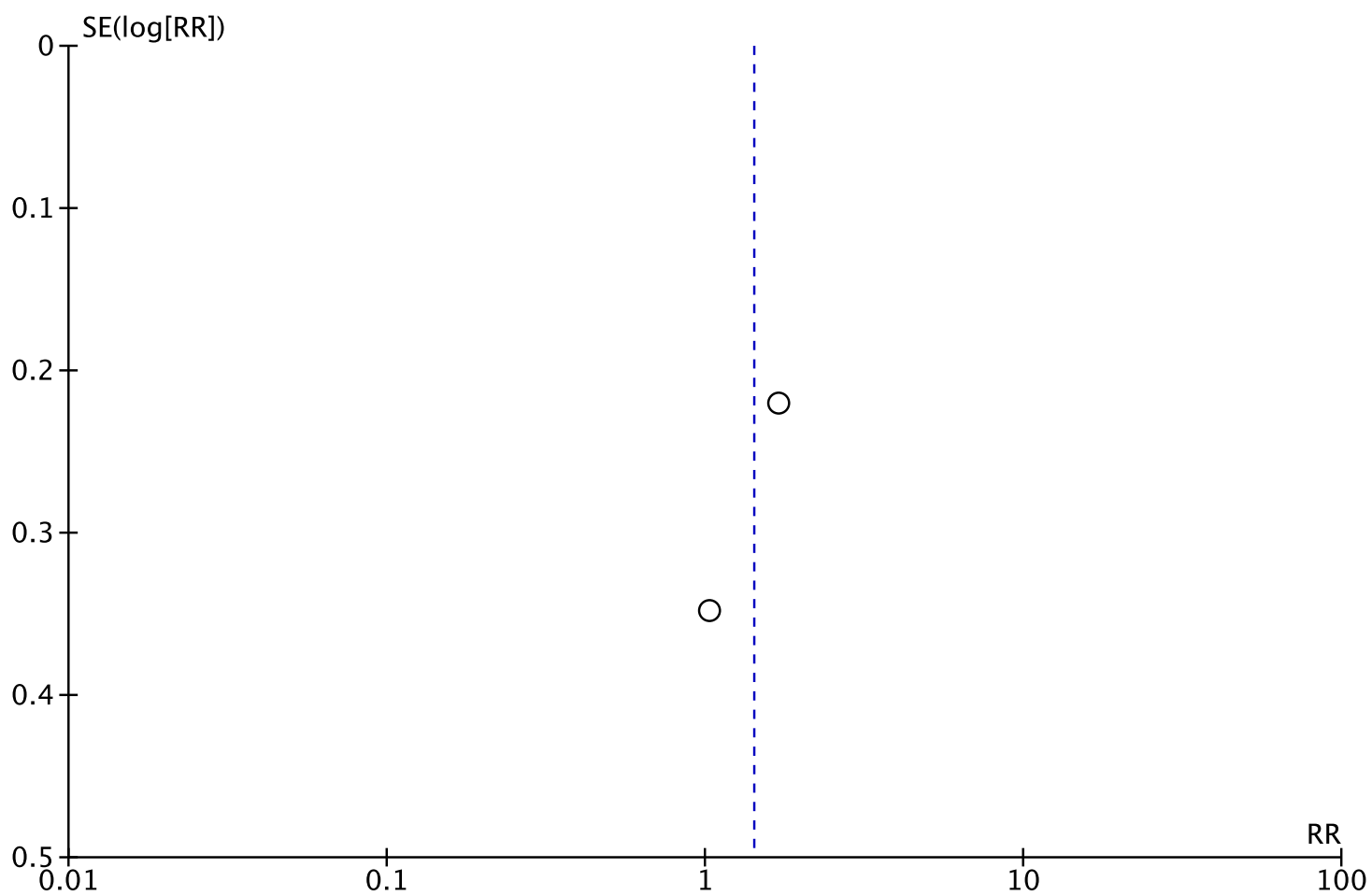

Supplement: Supplementary file 2 — Supplementary file2 Figure S1 Funnel plot for the co-primary outcome (susceptibility to COVID-19 infection) (PDF 19 KB) [file 43032_2024_1707_MOESM2_ESM.pdf]
